# A Blissful Role of Probiotic Therapy as an Adjunct to Periodontal Surgery in the Treatment of Periodontitis

**DOI:** 10.7759/cureus.71180

**Published:** 2024-10-10

**Authors:** Banashree Baishya, Sweta Yadav, Kajal Mahajan, Pratiksha Kumar, Hasbeena Ali, Mallika Sethi Rishi, Pratibha Wankhede

**Affiliations:** 1 Periodontics, Hi-Tech Dental College and Hospital, Bhubaneswar, IND; 2 Periodontology, Teerthanker Mahaveer Dental College and Research Centre, Teerthanker Mahaveer University, Moradabad, IND; 3 Periodontology, Ram Krishna Dharmarth Foundation University Dental College and Research Center, Bhopal, IND; 4 Oral Pathology and Microbiology, Government College of Dentistry, Indore, IND; 5 Periodontology, Dental House Oral Healthcare Centre, Vattamkulam, Kerala, IND; 6 Periodontology, Inderprastha Dental College and Hospital, Ghaziabad, IND; 7 Public Health, Shalinitai Meghe College of Nursing, Datta Meghe Institute of Higher Education and Research, Wardha, IND

**Keywords:** chronic periodontitis, clinical parameters, gingival index (gi), open flap debridement (ofd), probing pocket depth (ppd), probiotics, relative attachment level (ral)

## Abstract

Background

Periodontitis is a chronic inflammatory disease that alters the alveolar bone structure, requiring treatment ranging from non-surgical to surgical periodontal therapies based on its severity. Surgical interventions, such as the modified Widman flap procedure and the open flap technique combined with methods like platelet-rich fibrin (PRF), guided tissue regeneration (GTR), and bone grafts, aim to reduce periodontal pockets and regenerate lost tissues. The presence of pathogenic bacteria and the absence of beneficial bacteria contribute to periodontitis, with probiotics-live microorganisms that offer health benefits emerging as a promising adjunct in periodontal therapy. Probiotics can inhibit harmful organisms and enhance the oral mucosal lining, potentially improving clinical outcomes when used alongside surgical procedures. This study aims to evaluate the efficacy of combining probiotics with open flap debridement (OFD) in managing chronic periodontitis, comparing it to OFD alone.

Methodology

Eighty individuals were recruited in this double-blind, randomization clinically controlled, split-mouth trial. The enrolled individuals were categorized in a random manner into either Group A (OFD) or Group B (OFD with probiotic therapy, OFD + P).

Results

Based on the results, significant differences were observed between the OFD and OFD + P groups. At baseline, the mean probing pocket depth (PPD) was 6.49 mm in the OFD group and 5.68 mm in the OFD + P group. After three weeks, the PPD decreased to 4.71 mm in the OFD group and 3.95 mm in the OFD + P group, with a p-value of 0.021, indicating a significant difference. By 12 weeks, the PPD was 3.20 mm for OFD and 2.74 mm for OFD + P, though the difference was not statistically significant (p-value 0.108). For relative attachment level (RAL), a significant difference was noted after three weeks with a p-value of 0.018; however, differences at baseline and 12 weeks were not significant. The gingival index (GI) did not show significant differences between the groups at any time point. Within-group analyses revealed significant improvements in PPD, RAL, and GI for both groups over time, with all p-values < 0.001. The OFD + P group demonstrated superior outcomes compared to the OFD group in PPD and RAL after three weeks.

Conclusion

The use of probiotics in managing periodontal disease offers a cost-effective and convenient treatment option for periodontitis. Its integration into periodontal therapy should be emphasized for its potential benefits across all age groups, benefiting both periodontal surgeons and general dentists. Further research is needed to understand how well probiotics persist in the oral microflora and their precise effects on periodontal health. The future of periodontal therapy could greatly benefit from probiotics as a natural, food-based approach to enhancing immunity and improving oral health.

## Introduction

As periodontitis is a chronic disease with an inflammatory origin, it causes significant alterations in the normal structure of the alveolar process. However, these changes vary from person to person depending on the severity and extent of the disease, necessitating treatments that range from non-surgical to surgical periodontal therapy. Surgical therapy often becomes essential for a periodontal surgeon to access previously unreachable areas, enhance the reduction or elimination of periodontal pockets, and restore lost periodontal tissues through processes known as new attachment or periodontal regeneration [1,2]. When all factors are considered, this approach fulfills the true purpose of periodontal therapy. Another crucial factor is selecting the appropriate type of periodontal therapy. Numerous surgical techniques are available, including the modified Widman flap procedure, the open flap technique in combination with platelet-rich fibrin (PRF), guided tissue regeneration (GTR), bone grafts, probiotics, and more [1,3,4].

The possibility of tissue regeneration increases if intrabony defects are adequately addressed [2,5]. Research shows that the development of periodontal disease involves multiple factors, starting with the host's immune response and bacterial challenge. The presence of pathogenic bacteria and the absence of beneficial bacteria, combined with the host's susceptibility, are key factors in the onset of periodontitis [6]. Despite this understanding, the primary focus of treatment remains the reduction or elimination of periodontal pathogens [3]. Probiotics, defined as "live microorganisms which, when administered in adequate amounts, confer a health benefit on the host" by the World Health Organization (WHO), play an important role in periodontal therapy [7].

Probiotic preparations can bind to the biofilm on tooth structures, forming a protective barrier for the mucosal lining and shielding it from oral diseases [7,8]. This probiotic-coated biofilm prevents harmful organisms from occupying spaces that would otherwise become protective niches for future periopathogens [4]. As a result, the use of beneficial bacteria, in the form of prebiotics or probiotics, offers a promising adjunct to surgical periodontal therapy [8]. In addition to their antimicrobial properties, probiotics exhibit anti-inflammatory effects, introducing a unique approach to enhancing the presence of beneficial microorganisms in the oral cavity [9]. Although the health benefits of probiotics through oral administration or local delivery have been widely explored, data regarding their application during periodontal surgery remains limited [9]. It is hypothesized that incorporating probiotics during periodontal surgery will improve clinical outcomes in patients with chronic periodontitis. The primary objective of this research is to evaluate and compare the effects of open flap debridement (OFD) with and without the use of a combined probiotic formulation in the treatment of chronic periodontitis.

## Materials and methods

Study design and setting

**Figure 1:**
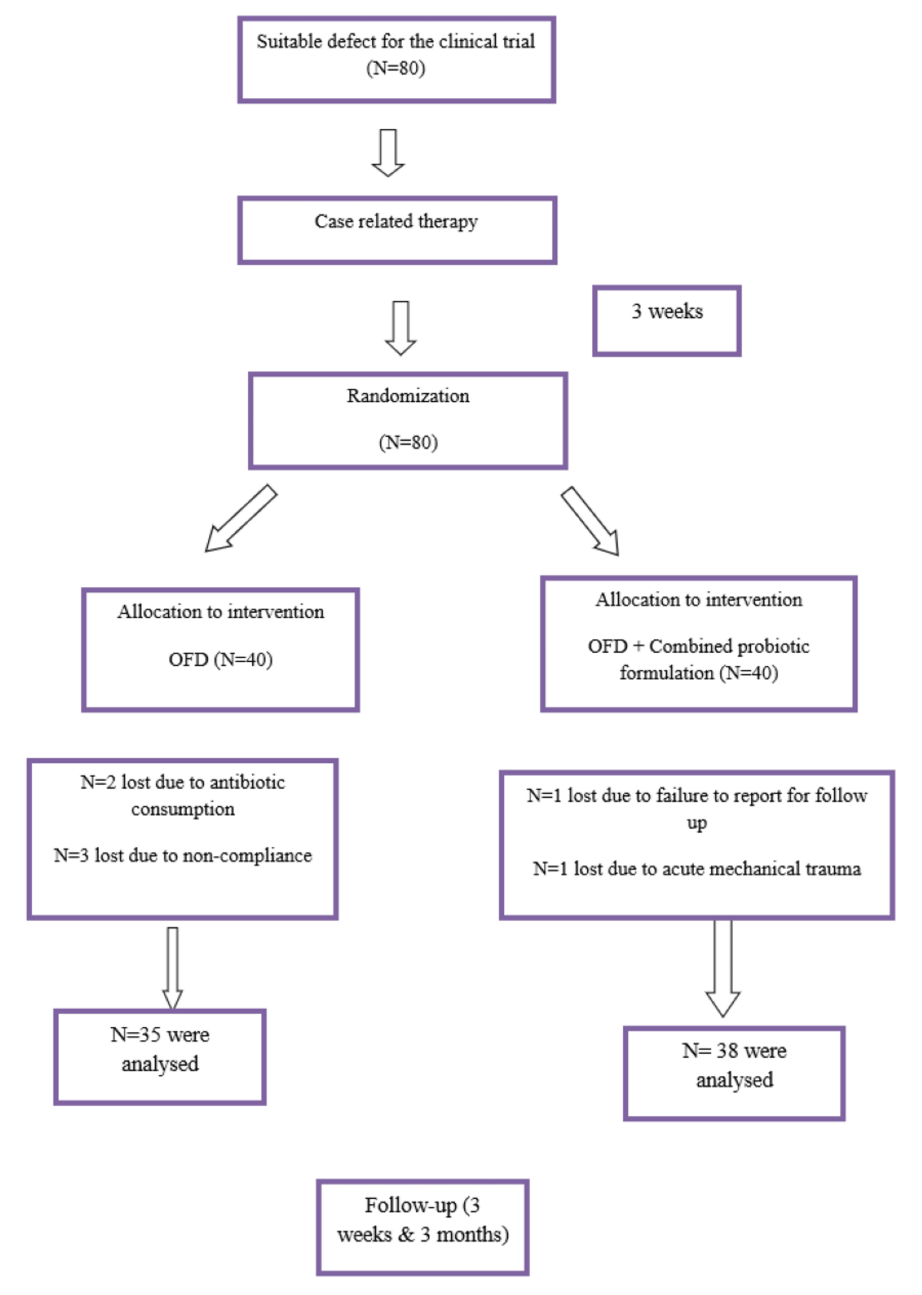
Consolidated Standards of Reporting Trials (CONSORT) flow diagram

This randomized, clinical controlled trial, parallel, prospective, double-blind, longitudinal study was approved by the Institutional Review Board. Patients were referred to the Department of Periodontology and were screened for the study. Informed permission was voluntarily obtained from all the participants with bilateral periodontal pockets prior to their enrolment into the clinical trial. Figure 1 shows the Consolidated Standards of Reporting Trials (CONSORT) flow diagram.

Selection Criteria

The inclusion criteria for this study involved male or female patients who were healthy and without any systemic illness. Participants were required to have a minimum of three natural teeth in each quadrant, excluding third molars, and must have presented with chronic periodontitis, with periodontal pockets larger than 6 millimeters (mm) and intrabony defects bilaterally. Patients were selected after at least six weeks post-conventional periodontal therapy and had to be 18 years or older, with pocket depths of 6 mm or greater and relative attachment levels of 10 mm or more. Additionally, a minimum of 3 mm of keratinized gingival surface was required, and patients with dental caries or endodontic-related issues were excluded. Subjects could not have undergone periodontal surgery or taken antibiotics or probiotics in the past six months. The exclusion criteria ruled out patients with furcation defects, pregnant or lactating women, and those with poor oral hygiene or compliance. Individuals with systemic disorders such as diabetes mellitus, rheumatoid arthritis, renal disease, or neurologic or immunologic conditions that might affect periodontal health or healing were also excluded. Patients who had used medications like non-steroidal anti-inflammatory drugs (NSAIDs), anticoagulants, or antibiotics in the past six months were not eligible, nor were smokers due to their known impact on periodontal disease and healing outcomes. Furthermore, individuals with acute oral lesions or necrotizing ulcerative periodontitis were not included in the study.

Data sources and variables

The probiotic product selected for the study was Bifilac‑Hp capsules, containing Lactobacillus sporogenes (100 million), Streptococcus faecalis T‑110JPC (60 million), Clostridium butyrium TO‑A (four million), and Bacillus mesentericus TO‑A JPC (two million). The combination of these probiotic strains acts synergistically, enhancing the likelihood of permanent attachment regeneration. The capsules were ground into a powdered form to be inserted into osseous defects in conjunction with the OFD procedure. Eighty patients with chronic periodontitis, aged between 30 and 45 years, were recruited for the study without gender discrimination. Participants met the following criteria: they were not undergoing dental treatment at the time, had not been exposed to antibiotics for the past three months, and had not consumed probiotic supplements during that period. To assign defects to either the test or control group, a coin toss method was used, and the statistician was blinded to the surgical procedures and measurements. Clinical parameters were recorded using a UNC-15 probe (Hu-Friedy Manufacturing, Co., LLC, Chicago, Illinois, United States) at baseline (BL), three weeks, and three months post-treatment. To standardize probe positioning, occlusal stents were fabricated using cold-cure acrylic resin, and millimeter measurements were rounded to the nearest 0.5 mm. The parameters recorded were the gingival index (GI) (Loe and Silness, 1963), probing depth (PD), which measured from the gingival margin to the base of the periodontal pocket, and relative attachment level (RAL), from a fixed point on the stent to the base of the pocket.

The study was designed as a double-blind, clinically controlled, randomized trial over a three-month period. Participants were evenly divided into two groups: Group A (40 subjects) as the control group and Group B (40 subjects) as the test/probiotic group. The control group underwent OFD, while the test group received OFD with the addition of powdered probiotics into the osseous defects. Sutures were placed, and postoperative instructions were provided. Patients were recalled for suture removal after seven days, and for reevaluation after three weeks and three months. During the study, two participants were lost from Group A due to antibiotic use, and three others were lost due to non-compliance. Similarly, two participants from Group B were lost due to irregular follow-up and acute mechanical trauma. As a result, 73 subjects (35 in the control group and 38 in the test group) were analyzed.

Following local anesthesia, crevicular incisions were made, extending from one tooth mesially and another distally from the osseous defect zone, without vertical incisions. A full-thickness flap was elevated, and debridement of granulation tissue from the root surfaces in both groups was carried out using manual and ultrasonic instruments. The defect types were rechecked and confirmed before being randomly assigned to either OFD alone or OFD combined with probiotic administration. The area was cleaned and dried using an aspirator and cotton gauze, after which the probiotic was applied to fill the defect up to the crestal margin in the test group. The flaps were approximated using silk sutures, and the treated area was covered with a periodontal dressing. NSAIDs were advised before and four hours after surgery. Patients were instructed to avoid interdental cleaning for the first four postoperative weeks. In addition, a 0.2% chlorhexidine-digluconate mouthwash was prescribed for two weeks to maintain oral hygiene and reduce microbial load. Sutures were removed seven days post-surgery, and follow-up appointments were scheduled for reevaluation at three weeks and three months, during which the same protocol for recording clinical parameters was repeated to assess postoperative changes.

Statistical analysis

The data in the study was presented as means with standard deviations for probing pocket depth (PPD), RAL, and GI. To assess differences between OFD and OFD combined with probiotics (OFD + P) groups, statistical significance was evaluated using independent t-tests for comparisons at baseline, three weeks, and 12 weeks. Intra-group comparisons were performed using paired t-tests to examine changes over time within each group.

## Results

Table 1 illustrates the comparison of PPD between the OFD and OFD + P groups. At baseline, the mean PPD was 6.49 mm for the OFD group and 5.68 mm for the OFD + P group. After three weeks, the PPD decreased to 4.71 mm in the OFD group and 3.95 mm in the OFD + P group. By 12 weeks, the PPD was 3.20 mm in the OFD group and 2.74 mm in the OFD + P group. Statistically significant differences were observed between the groups at baseline and after three weeks, with p-values of 0.049 and 0.021, respectively.

**Table 1:** Comparison of PPD between OFD and OFD+P groups PPD: probing pocket depth; OFD: open flap debridement; OFD + P: open flap debridement combined with probiotics; mm: millimeters Independent t-test applied. p-value < 0.05 considered to be statistically significant

PPD	Group	N	Mean (mm)	Standard deviation (mm)	Standard error mean	F	95% CI		p-value
							Lower	Upper	
At Baseline	OFD	35	6.49	1.69	0.29	0.05	0.00	1.60	0.049
	OFD + P	38	5.68	1.73	0.28				
After 3 weeks	OFD	35	4.71	1.41	0.24	0.01	0.12	1.42	0.021
	OFD + P	38	3.95	1.37	0.22				
After 12 weeks	OFD	35	3.20	1.32	0.22	0.98	-0.10	1.03	0.108
	OFD + P	38	2.74	1.11	0.18				

Table 2 compares the RAL between the OFD and OFD + P groups. At baseline, the mean RAL was 9.54 mm in the OFD group and 8.50 mm in the OFD + P group. After three weeks, the RAL decreased to 7.69 mm in the OFD group and 6.76 mm in the OFD + P group. By 12 weeks, the RAL was 6.03 mm in the OFD group and 6.97 mm in the OFD + P group. Significant differences were noted between the groups after three weeks, with a p-value of 0.018, while differences at baseline and 12 weeks were not statistically significant.

**Table 2:** Comparison of RAL between OFD and OFD+P groups OFD: open flap debridement; OFD + P: open flap debridement combined with Probiotics; RAL: relative attachment level; mm: millimeters Independent t-test applied. p-value < 0.05 considered to be statistically significant

RAL	Group	N	Mean (mm)	Standard deviation (mm)	Standard error mean	F	95% CI		p-value
							Lower	Upper	
At Baseline	OFD	35	9.54	2.32	0.39	0.30	-0.04	2.13	0.060
	OFD + P	38	8.50	2.33	0.38				
After 3 weeks	OFD	35	7.69	1.57	0.26	0.63	0.16	1.70	0.018
	OFD + P	37	6.76	1.69	0.28				
After 12 weeks	OFD	35	6.03	1.76	0.30	1.36	-4.45	2.56	0.593
	OFD + P	38	6.97	10.27	1.67				

Table 3 presents the comparison of the GI between the OFD and OFD + P groups. At baseline, both groups had a mean GI of 2.00. After three weeks, the GI decreased to 0.49 in the OFD group and 0.76 in the OFD + P group. By 12 weeks, the GI was 0.34 in the OFD group and 0.37 in the OFD + P group. No significant differences were found between the groups at any time point.

**Table 3:** Comparison of the gingival index (GI) between OFD and OFD+P groups OFD: open flap debridement; OFD + P: open flap debridement combined with probiotics; GI: gingival index Independent t-test applied. p-value < 0.05 considered to be statistically significant

GI	Group	N	Mean	Standard deviation	Standard error mean	F	95% CI		p-value
							Lower	Upper	
At Baseline	OFD	35	2.00	0.00	0.00	NA	NA	NA	NA
	OFD + P	38	2.00	0.00	0.00				
After 3 weeks	OFD	35	0.49	0.56	0.10	0.27	-0.57	0.01	0.06
	OFD + P	37	0.76	0.68	0.11				
After 12 weeks	OFD	35	0.34	0.48	0.08	0.20	-0.25	0.20	0.82
	OFD + P	38	0.37	0.49	0.08				

Table 4 details the intra-group comparisons for clinical parameters in the control (OFD) group. For PPD, the mean decreased from 6.49 mm at baseline to 4.71 mm after three weeks and further to 3.20 mm after 12 weeks, with all comparisons showing statistically significant differences (p-values < 0.001). For RAL, the mean decreased from 9.54 mm at baseline to 7.69 mm after three weeks and to 6.03 mm after 12 weeks, with significant changes observed (p-values < 0.001). For GI, the mean reduced from 2.00 at baseline to 0.49 after three weeks and 0.34 after 12 weeks, with all comparisons being statistically significant (p-values < 0.001).

**Table 4:** Intra-group comparison of the clinical parameters of the control (OFD) group PPD: probing pocket depth; OFD: open flap debridement; RAL: relative attachment level; GI: gingival index; mm: millimeters Paired t-test applied. p-value < 0.05 considered to be statistically significant

		Mean	N	Standard deviation	Standard error mean	p-value
Pair 1	PPD at Baseline	6.49 mm	35.00	1.69 mm	0.29	0.000
	PPD after 3 weeks	4.71 mm	35.00	1.41 mm	0.24	
Pair 2	PPD at Baseline	6.49 mm	35.00	1.69 mm	0.29	0.000
	PPD after 12 weeks	3.20 mm	35.00	1.32 mm	0.22	
Pair 3	PPD after 3 weeks	4.71 mm	35.00	1.41 mm	0.24	0.000
	PPD after 12 weeks	3.20 mm	35.00	1.32 mm	0.22	
Pair 4	RAL at Baseline	9.54 mm	35.00	2.32 mm	0.39	0.000
	RAL after 3 weeks	7.69 mm	35.00	1.57 mm	0.26	
Pair 5	RAL at Baseline	9.54 mm	35.00	2.32 mm	0.39	0.000
	RAL after 12 weeks	6.03 mm	35.00	1.76 mm	0.30	
Pair 6	RAL after 3 weeks	7.69 mm	35.00	1.57 mm	0.26	0.000
	RAL after 12 weeks	6.03 mm	35.00	1.76 mm	0.30	
Pair 7	GI at Baseline	2.00	35.00	0.00	0.00	0.000
	GI after 3 weeks	0.49	35.00	0.56	0.10	
Pair 8	GI at Baseline	2.00	35.00	0.00	0.00	0.000
	GI after 12 weeks	0.34	35.00	0.48	0.08	
Pair 9	GI after 3 weeks	0.49	35.00	0.56	0.10	0.000
	GI after 12 weeks	0.34	35.00	0.48	0.08	

Table 5 provides the intra-group comparisons for the test (OFD + P) group. The mean PPD decreased from 5.68 mm at baseline to 3.95 mm after 3 weeks and to 2.74 mm after 12 weeks, with all differences being statistically significant (p-values < 0.001). For RAL, the mean reduced from 8.46 mm at baseline to 6.76 mm after 3 weeks and 6.97 mm after 12 weeks, with all changes showing significant differences (p-values < 0.001). For GI, the mean decreased from 2.00 at baseline to 0.76 after 3 weeks and 0.37 after 12 weeks, with statistically significant changes (p-values < 0.001).

**Table 5:** Intra-group comparison of the clinical parameters of the test (OFD+P) group PPD: probing pocket depth; OFD + P: open flap debridement combined with probiotics; RAL: relative attachment level; GI: gingival index; mm: millimeters Paired t-test applied. p-value < 0.05 considered to be statistically significant

		Mean	N	Standard deviation	Standard error mean	p-value
Pair 1	PPD at Baseline	5.6842 mm	38	1.72588 mm	0.27997	0.000
	PPD after 3 weeks	3.9474 mm	38	1.37443 mm	0.22296	
Pair 2	PPD at Baseline	5.6842 mm	38	1.72588 mm	0.27997	0.000
	PPD after 12 weeks	2.7368 mm	38	1.10733 mm	0.17963	
Pair 3	PPD after 3 weeks	3.9474 mm	38	1.37443 mm	0.22296	0.000
	PPD after 12 weeks	2.7368 mm	38	1.10733 mm	0.17963	
Pair 4	RAL at Baseline	8.4595 mm	37	2.35224 mm	0.38671	0.000
	RAL after 3 weeks	6.7568 mm	37	1.68993 mm	0.27782	
Pair 5	RAL at Baseline	8.5000 mm	38	2.33366 mm	0.37857	0.000
	RAL after 12 weeks	6.9737 mm	38	10.26799 mm	1.66569	
Pair 6	RAL after 3 weeks	6.7568 mm	37	1.68993 mm	0.27782	0.000
	RAL after 12 weeks	7.0000 mm	37	10.40833 mm	1.71112	
Pair 7	GI at Baseline	2.0000	38	0.00000	0.00000	0.000
	GI after 3 weeks	0.7632	38	.67521	0.10953	
Pair 8	GI at Baseline	2.0000	38	0.00000	0.00000	0.000
	GI after 12 weeks	0.3684	38	.48885	0.07930	
Pair 9	GI after 3 weeks	0.7632	38	.67521	0.10953	0.000
	GI after 12 weeks	0.3684	38	.48885	0.07930	

## Discussion

The present trial is designed to evaluate the efficacy of OFD + P versus OFD alone in patients suffering from chronic periodontal disease. Results indicate significant improvement in the test group (OFD + P) concerning the RAL and PPD at the three-week postsurgical reevaluation. These findings align with those of Teughels et al., who demonstrated similar clinical improvements following the use of probiotic lozenges alongside traditional periodontal treatment at three, six, nine, and 12 weeks [3,10,11]. Similarly, Shimauchi et al. reported improvements in clinical parameters after the intake of freeze-dried Lactobacillus salivarius WB21-containing tablets over a four-to-eight-week period [12]. Moreover, Tekce et al. found that lozenges containing Lactobacillus reuteri, when administered as an adjunct to scaling and root planing (SRP), produced comparable outcomes to those observed in the current study after 21, 90, 180, and 360 days [13]. The significant clinical improvements seen in this trial's test group may be attributed to the beneficial role probiotics play in modulating the oral microbiome and reducing the inflammatory response. Probiotics have been found to inhibit the colonization of pathogenic bacteria by competing for adhesion sites and nutrients while promoting the growth of beneficial microorganisms. This competition creates a more balanced oral environment, which could explain the reduction in PPD and improved attachment levels observed in the test group. Several studies, including those by Hardan et al. and Comelli et al., have highlighted probiotics' ability to reduce pathogenic bacterial loads and modulate host immune responses in the oral cavity [14,15].

However, it is crucial to note that while clinical improvements were significant in the early stages (three-week postsurgical evaluation), the differences between the test group and the control group (OFD alone) diminished over time. By the 12-week evaluation, there was no statistically significant difference between the two groups in terms of RAL and PPD. This is in contrast to the findings of Teughels et al. [3] and Tekce et al. [13], who reported sustained improvements up to the 12th week following combined probiotic application. This discrepancy could be due to variations in study design, including differences in probiotic strains, dosage, and method of delivery. The present study involved the application of probiotics directly at the surgical site, while the other studies primarily utilized oral probiotic lozenges or tablets. The direct delivery method used in this trial may have resulted in a more immediate, but shorter-lived, effect compared to the sustained release of probiotics through oral administration. When evaluating gingival inflammation, the test group (OFD + P) demonstrated significantly better results than the control group (OFD alone) after three weeks. These findings are consistent with the anti-inflammatory properties of probiotics, as documented in the work of Shimauchi et al. [12], Tekce et al. [13], and Widyarman et al. [16]. Probiotics have been shown to exert anti-inflammatory effects by modulating cytokine profiles, thereby reducing the host's inflammatory response to periodontal pathogens. However, by the 12-week evaluation, the intergroup differences in gingival inflammation were no longer statistically significant. This suggests that while probiotics can offer short-term benefits in reducing inflammation, their long-term effects may be limited unless supported by sustained probiotic use.

An interesting observation in this study is the intra-group comparison. Both the test group (OFD + P) and the control group (OFD alone) showed significant improvements in PPD, RAL, and GI at three and 12 weeks compared to baseline values. These findings reflect the efficacy of OFD as a standard surgical treatment for periodontitis. OFD allows for improved access to the root surfaces and periodontal pockets, facilitating thorough debridement and removal of microbial deposits, which leads to clinical improvement. The combination of OFD with probiotics enhanced these clinical outcomes, particularly in the early postoperative period, likely due to the probiotics' ability to accelerate healing and reduce inflammation. It is also worth noting that while the present study incorporated a surgical intervention (OFD), previous studies by Teughels et al. [3], Tekce et al. [13], and Shimauchi et al. [12] focused on non-surgical approaches, primarily using probiotics in lozenge or tablet form. This difference in approach raises important considerations about the role of probiotics in conjunction with various periodontal therapies. The present findings suggest that probiotics may offer additional short-term benefits when combined with surgical interventions, though their long-term efficacy remains uncertain.

Limitations of the study

The present study has several limitations that should be acknowledged. First, the sample size was relatively small, which may limit the generalizability of the findings to a broader population. Additionally, the follow-up period was limited to 12 weeks, preventing the assessment of the long-term effects of OFD + P. Variability in patient compliance with postoperative care and probiotic use could have also impacted the results. Furthermore, the study only focused on specific probiotic strains, and the potential effects of other strains remain unexplored.

## Conclusions

The use of probiotics in managing periodontal disease represents a significant advancement in the treatment of periodontitis. Probiotics are not only cost-effective but also convenient for enhancing periodontal therapy. Their application should be emphasized across all age groups, as they offer substantial benefits in both prevention and treatment. It is essential for periodontal surgeons and general dentistry practitioners to consider probiotics due to their compatibility with the human oral microbiome. Further research is needed to understand the survival of probiotic formulations in the oral environment and to explore their potential benefits. The future prospects of incorporating probiotics into periodontal surgery suggest a promising approach for improving periodontal health through natural immune modulation. The goal should be to prioritize dietary interventions over supplements or medications, aligning with a holistic approach to periodontal care.
